# Correlating the Effects of UV Aging on the Macro-Micro Behaviors of Asphalt with Its Molecular Mechanisms

**DOI:** 10.3390/ma18102165

**Published:** 2025-05-08

**Authors:** Han Xi, Lingyun Kong, Shixiong Hu, Songxiang Zhu

**Affiliations:** 1 College of Traffic Engineering, Huanghe Jiaotong University, Jiaozuo 454950, China; 18303863302@163.com (H.X.); shu@esu.edu (S.H.); 2 School of Civil Engineering, Chongqing Jiaotong University, Chongqing 400074, China; zhusongxiang95@gmail.com; 3 National and Local Joint Laboratory of Traffic Civil Engineering Materials, Chongqing Jiaotong University, Chongqing 400074, China

**Keywords:** UV aging, rheological property, chemical composition, molecular weight and distribution, molecular structure, correlation analysis

## Abstract

UV radiation can change the internal molecular composition, macroscopic rheological properties, and microscopic chemical composition of asphalt. To study the effect of ultraviolet aging on asphalt and its structure–activity relationship, its rheological properties were measured by dynamic shear rheology and multiple stress recovery creep tests, its chemical compositions were measured by component composition, elemental composition, and infrared spectrum tests, and its molecular weight, distribution, and molecular structure were determined by gel permeation chromatography and nuclear magnetic resonance tests. Then, the molecular weight and molecular structure, rheological properties, and microchemical aging behavior of asphalt after UV aging were characterized by correlation analysis, and the structure–activity relationship was analyzed. The results show that the deformation resistance and elastic recovery ability of asphalt after UV aging are enhanced, and the flow performance is decreased. The ultraviolet radiation caused the aromatic hydrocarbons containing naphthenes and long alkyl chains in the asphalt to break and connect with asphaltenes with a ring structure. The asphaltene content in each bitumen sample exceeded 46%, and that in KL reached 55%, indicating that the bitumen changed into a gel structure. UV aging causes the aggregation of asphalt molecules, and the aggregation of molecules narrows the molecular distribution boundary and moves in the direction of macromolecules, resulting in the reduction of the dispersion coefficient by 2–10%. Hydrogen atoms will undergo condensation and substitution reactions due to long-chain breaking, cyclization, or aromatization under UV action, and the breaking of C=C bonds in carbon atoms will increase the stable aromatic ring, strengthen the stiffness of the molecular backbone, and make it difficult for the backbone to spin. Through correlation analysis, it was found that the molecular composition index could characterize the aging behavior index of asphalt, and that the aromatic structure was the most critical molecular change. Further, it was found that the sulfoxide group and carbonyl group could be used as evaluation criteria for the UV aging of asphalt because the correlation between them was above 0.7. This study provides an essential index reference for evaluating the performance change of asphalt under ultraviolet aging to save testing time. Moreover, the molecular structure characterization revealed the changes in internal molecular composition that were behind the observed aging properties, providing a theoretical basis for research on asphalt anti-aging technology.

## 1. Introduction

The medium and long waves in the ultraviolet spectrum possess a potent penetrative capability, facilitating a photochemical reaction in asphalt when it is exposed to ultraviolet radiation. This reaction, combined with additional environmental factors such as oxygen, heat, and moisture, induces an aging phenomenon in asphalt [1,2,3]. The existing research indicates that, although the layer of asphalt affected by ultraviolet aging is approximately 10 μm, this depth corresponds to the thickness of the asphalt film used in road construction; thereby, UV aging significantly impacts the integrity of roads [4,5]. Notably, ultraviolet-aged asphalt pavement develops fine, dense cracks, allowing ultraviolet aging to penetrate more deeply into the pavement under the influence of rain, load, and oxygen. This deep-seated degradation can damage the underlying layers of the pavement, leading to a decline in the performance and operational quality of the asphalt pavement [6,7,8]. Asphalt aging primarily occurs through two mechanisms: ultraviolet aging and thermal oxidative aging. While thermal oxidative aging has been extensively researched with established simulation techniques and standardized protocols [9,10], it cannot be considered interchangeable with ultraviolet aging due to differing chemical bond scission processes and resultant outcomes [11,12].

The current body of research on the ultraviolet aging of asphalt primarily focuses on alterations in its macro and micro properties, as well as compositional changes. Li et al. examined the physical and rheological properties of SK-70 asphalt, finding that the softening point, viscosity, and complex modulus increased post-ultraviolet aging, while the ductility markedly decreased [13]. Eltwati et al. investigated the high- and low-temperature rheological properties of recycled asphalt and asphalt mixtures under ultraviolet aging, discovering that both the high- and low-temperature rheological properties of the mixtures were diminished by UV aging, with more severe degradation occurring in low-temperature rheological properties [14]. Li et al. conducted a chemical functional group analysis on the aging depth of asphalt under varying UV intensities, revealing that asphalt aging predominantly occurs in the initial stages, with a higher UV intensity producing a more substantial aging effect than concurrent UV exposure [4,15]. Zeng et al. explored the aging impact of ultraviolet light on asphalt at different depths using a stripping method, analyzing its rheological properties and component compositions, and determined that ultraviolet aging can penetrate up to 2200 μm [5]. Celauro et al. performed ultraviolet aging tests on two distinct grades of asphalt that are commonly utilized in Italy in order to study functional group changes, and observed degradation of the asphalt under UV light that lead to the formation of numerous oxygen-containing groups [16]. Zhang et al. evaluated the rheological and chemical functional group indexes of UV-aged asphalt under diverse environments, finding minimal differences in the rheological indexes between field and laboratory aging, but significant disparities in chemical functional group indexes [17]. Hou et al. used spectrophotometry to examine the changes in chemical functional groups that resulted from the ultraviolet aging of asphalt from five different oil sources, concluding that the observed change in absorbance during aging stemmed from an increase in asphaltenes [18]. Rajib et al. conducted thermal oxygen aging and ultraviolet aging tests on matrix asphalt and rubber asphalt, respectively, discovering that the rheological hardening of asphalt decreased under the influence of thermal oxygen and prolonged UV aging [19]. Yu et al. assessed the rheological properties of SBS asphalt at high and low temperatures under UV exposure, noting enhanced rutting resistance post-UV aging but increased rigidity and brittleness, which rapidly lead to pavement cracking at low temperatures [20]. Li et al. investigated the impact of UV–oxygen coupled aging on the rheological properties and functional groups of asphalt, highlighting the significant role of oxygen in amplifying the area and peak values of characteristic carbon-based functional groups [3]. However, consensus on the effects of UV aging and its evaluation criteria remains elusive [21,22], and the mechanism behind the molecular deconstruction and remodeling that occur during ultraviolet aging is still not fully understood.

This study aims to investigate the effects of ultraviolet aging on the rheological properties, chemical composition, and molecular structure of asphalt using four substrates that are commonly used in engineering. The analysis of these factors will provide performance indicators for asphalt at different scales and reveal patterns of molecular structural changes. Furthermore, the grey relational analysis method will be used to establish a structure–activity relationship between the ultraviolet aging behavior indicators of asphalt and its molecular structure. This research will serve as an essential index reference for evaluating performance changes in asphalt under ultraviolet aging and save testing time. Additionally, the molecular structure characterization will reveal the changes in internal molecular composition that are behind the aging properties of asphalt, providing a theoretical basis for research on asphalt anti-aging technology.

## 2. Materials and Test Method

### 2.1. Asphalt Material

Different types of KunLun AH-70 (KL), TaiPuKe AH-70 (TPK), ZhongHai AH-70 (ZH), and DongHai AH-70 (DH) bitumen were selected as the research objects. Their basic physical properties were determined by Technical Specifications for Highway Asphalt Pavement Construction (JTG F40-2017) [23] and Test Regulations for Highway Asphalt and Asphalt Mixtures (JTG E20-2011) [24]. The results are shown in Table 1.

### 2.2. Ultraviolet Aging Test

**Figure 1:**
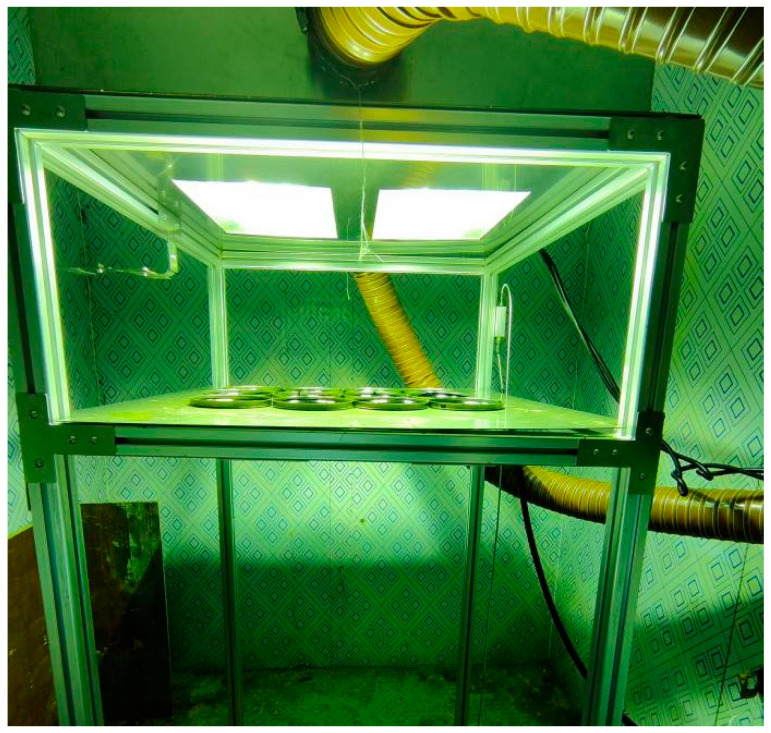
UV aging chamber interior diagram.

A 500 W high-pressure mercury lamp was used as an ultraviolet aging light source to simulate outdoor ultraviolet aging, as shown in Figure 1. Ultraviolet aging was carried out using an all-weather 24 h uninterrupted aging mode. The wavelength of ultraviolet light ranges from 280 to 400 nm, with an irradiation intensity of 65 w/m^2^. To guarantee the ultraviolet intensity, the aging sample was placed 50 cm away from the ultraviolet lamp. The aging time was 2 d, 4 d, 8 d, 10 d, and 16 d, and the aging temperature was 60 °C. Among the aging times, the 16-day aging is equivalent to the ultraviolet radiation in Chongqing area for one year, and the temperature of 60 °C is the highest temperature on the road surface in Chongqing area during summer. Because ultraviolet aging only acts on the asphalt surface, we determined that the sample should not be too thick, and so we selected a sample of 1 mm here. In the test, the aging tray had a diameter of 14 cm. According to the 1 mm aging thickness and the asphalt density, we ascertained that about 15.4 g of asphalt should be poured. Therefore, the mass of asphalt was be controlled between approximately 15.2 and 15.6 g by using a millage balance during pouring.

### 2.3. Rheological Performance Test

Dynamic shear rheological temperature scanning (TS) and multiple stress creep recovery tests (MSCR) were employed to determine the phase angle, rutting factor, and resilience of asphalt. The dynamic shear rheometer was utilized in both sets of test apparatuses. In the TS test, the set frequency was 10 rad/s, the disc rotor diameter was 25 mm, the parallel plate spacing was 1 mm, and the strain control mode strain was 1%. The MSCR test was conducted successively under two stresses of 0.1 kPa and 3.2 kPa, and the test temperature was 60 °C.

### 2.4. Chemical Composition Test and Its Index Calculation

#### 2.4.1. Chemical Composition Test

The asphalt chemical composition test entailed component and infrared spectrum tests. The test can determine the component composition, characteristic peak, and functional group of asphalt samples under different aging times. A rod TLC-hydrogen flame ion detector was used to carry out the component composition test. The solution was a dichloromethane–bituminous mixture with a 20 mg/mL bituminous concentration. The infrared spectrum test’s infrared spectrometer had a resolution of 4 cm^−1^, a scanning frequency of 64 times, and a test range of 400~4000 cm^−1^.

#### 2.4.2. The Component Content and Colloidal Instability Index Calculation

Asphalt can be divided into the following groups: saturate (*S*) and aromatic (*A_s_*) light components and resin (*R*) and asphaltene (*A_r_*) recombination components. The colloidal instability index (*I_c_*) represents the change in the colloidal structure of asphalt. The *I_c_* can be obtained by calculating the proportion of asphalt recombination components and that of light components, as shown in Formula (1) [25].

(1) $$I_{C}=\frac{A_{s}+S}{A_{r}+R}$$

where *I_c_* is the colloidal instability index, *A_r_* is the aromatic proportion, *R* is the resin proportion, *A_s_* is the asphaltene proportion, and *S* is the saturated proportion.

#### 2.4.3. The Sulfoxide and Carbonyl Functional Group Indices Calculation

According to the Lambert–Beer law, the characteristic peak area at 2000~6000 cm^−1^ was taken as a reference value [26]. Formulas were used to calculate the sulfoxide and carbonyl functional group indices (2) and (3) as well as to calculate the sulfoxide group index (*SI*) and carbonyl group index (*CI*).

(2) $$SI=\frac{A_{S=O}}{A_{2000\text{–}6000}}$$

(3) $$CI=\frac{A_{C=O}}{A_{2000\text{–}6000}}$$

where *SI* is the sulfoxide index; *A_S=O_* is the 1030 cm^−1^ characteristic peak area; *CI* is the carbon index; *A_C=O_* is the 1700 cm^−1^ characteristic peak area; *A*_2000–6000_ is the 2000~6000 cm^−1^ band total area.

### 2.5. Molecular Mechanism Test and Its Index Calculation

#### 2.5.1. Molecular Composition Test

The molecular weight and distribution of the asphalt were tested by gel permeation chromatography (GPC). In accordance with the procedure for gel permeation chromatography (GPC) using tetrahydrofuran as eluent (GB/T 21863-2008) [27], the test was performed using a gel chromatographic column and an aqueous gel column, and a chromatographic grade tetrahydrofuran was selected as a solvent; the concentration of the sample solution was 5 mg/mL, and the flow rate was 1 mL/min. A superconducting nuclear magnetic resonance spectrometer was used to measure the content and distribution of hydrogen and carbon atoms in the asphalt. The resonant frequency of the test was 400 MHz, the solvent was tritiated chloroform, and the internal standard was TMS (tetramethylsilane).

#### 2.5.2. Molecular Weight Calculation and Molecular Relative Mass Distribution

(1) Molecular weight calculation

The calculation methods for the asphalt number average molecular weight (*M_n_*), weight average molecular weight (*M_w_*), and dispersion coefficient (*D*) are shown in Formulas (4)–(6) [28].

(4) $$M_{n}=\frac{\sum N_{i}M_{i}}{\sum N_{i}}$$

(5) $$M_{w}=\frac{\sum W_{i}M_{i}}{\sum W_{i}}$$

(6) $$D=\frac{M_{w}}{M_{n}}$$

where *M_n_* is the number average molecular weight; *M_w_* is the weight average molecular weight; *D* is the dispersion coefficient; *M_i_* is the molecular weight; *N_i_* is the molecular weight of the number of molecules of *M_i_*; *W_i_* is the molecular weight of the component of *M_i_*.

(2) Molecular relative mass distribution

Taking the logarithm of the weight average relative molecular mass *M_w_* as the horizontal coordinate and the relative volume content of the asphalt as the vertical coordinate, the relative mass distribution diagram of the asphalt can be obtained. *W_F_*/d (Log*M_w_*) represents the differential curve graph of Log*M_w_* [29]. Since the horizontal and vertical coordinates in the figure are relative values, the coordinates are dimensionless.

#### 2.5.3. Molecular Structure Calculation

(1) Attribution of hydrogen and carbon atoms

**Figure 2:**
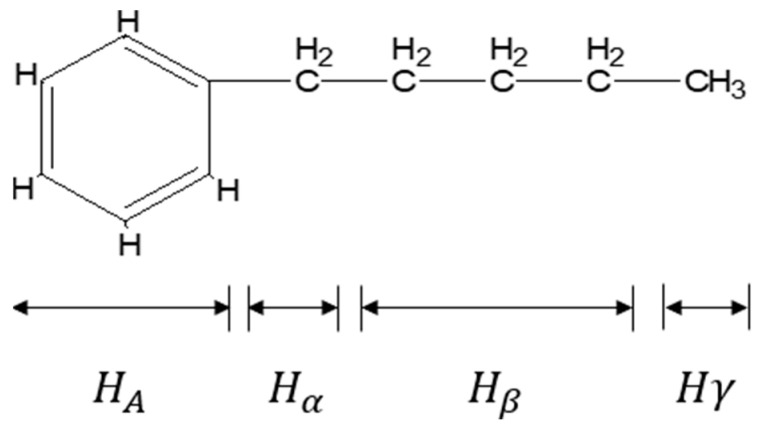
Schematic diagram of attribution of hydrogen atoms.

Hydrogen atoms can be divided into aliphatic hydrogen atoms (δ = 0.5~4.0) and aromatic hydrogen atoms (*H_A_*, δ = 6.0~9.0) according to the region in which they are located. The aliphatic hydrogen atoms can be further divided into *H_α_* (δ = 0.5~1.0), *H_β_* (δ = 1.0~2.0), and *H_γ_* (δ = 2.0~4.0) [30]. Asphalt carbon atoms mainly contain aromatic and fatty carbon [31]. The attribution of hydrogen and carbon atoms is shown in Table 2, and the schematic diagram of the attribution of hydrogen atoms is shown in Figure 2.

(2) Hydrogen spectra and carbon spectra

**Figure 3:**
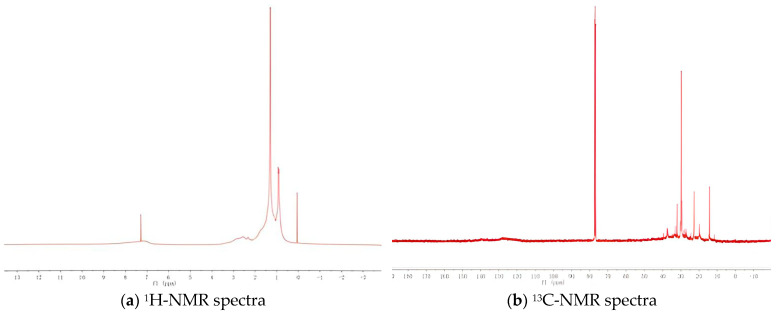
The hydrogen and carbon spectra of asphalt.

The hydrogen spectra (^1^H-NMR spectra) and carbon spectra (^13^C-NMR spectra) of the asphalt are obtained by taking the absorption peak position (chemical shift) of magnetic hydrogen nuclei in different chemical environments of the sample molecule as the horizontal coordinate (unit: ppm) and the relative height of the measured absorption peak (resonance signal strength) as the ordinate; since the ordinate is the relative height of the absorption peak, there is no rigidity, as shown in Figure 3.

(3) Carbon atom content

The improved Brown-Ladner method was used to determine the distribution of carbon atoms in asphalt [26]. Based on integrating the peak areas of the fatty carbon zone (*A_S_*) and aromatic carbon zone (*A_A_*) in the ^13^C-NMR spectra, the aromatic carbon atom content (*C_A_*), fatty carbon atom content (*C_N+P_*), and asphalt aromaticity (*f_A_*) of asphalt were calculated according to Formulas (7)–(9).

(7) $$C_{A}=\frac{A_{C_{A}}}{A_{C_{A}}+A_{C_{N+F}}}$$

(8) $$C_{N+F}=\frac{A_{C_{N+F}}}{A_{C_{A}}+A_{C_{N+F}}}$$

(9) $$f_{A}=\frac{A_{s}}{A_{A}+A_{s}}$$

where *C_A_* is the aromatic carbon atom content; *C_N+F_* is the fatty carbon atom content; *A_CA_* is the peak area of the aromatic carbon region; *A_CN+F_* is the peak area of the fatty carbon region; *A_A_* is the integral area of the aromatic carbon region; *A_S_* is the integral area of aromatic carbon region; *f_A_* is the asphalt aromaticity.

(4) Molecular structure parameter

The calculation is based on the content of aromatic carbon atoms and H_A_, H_α_, H_β_, and H_γ_ in the attribution of hydrogen elements. The condensation index of the aromatic ring system (*H_AU_*/*C_A_*), the degree of branching of the alkane chain (*BI*), and the peripheral hydrogen substitution rate (*σ*) of the aromatic ring system were calculated according to Formulas (10)–(12) [32].

(10) $$H_{AU}/C_{A}=\frac{H_{A}+H_{\alpha }/2}{C_{A}}$$

(11) $$BI=\frac{H_{\gamma }}{H_{\beta }}$$

(12) $$\sigma =\frac{H_{\alpha }/2}{H_{A}+H_{\alpha }/2}$$

where *H_AU_*/*C_A_* is the total hydrogen number; *BI* is the alkane chain branching degree; σ is thenaphthenic carbon number; *C_A_* is the aromatic carbon atom content; *H_A_*, *H_α_*, *H_β_*, and *H_γ_* are the same as in Table 2.

### 2.6. Relevance Calculation

The correlation degree is calculated by using mathematical software (SPSS 26.0) based on the grey correlation method, and its internal calculation logic is shown as follows.

(1) Reference series and comparative series

The reference sequence is composed of data that can represent the behavior characteristics of the system, and the comparison sequence is composed of data that impacts the reference sequence.

(13) $$X_{0}=\{X(1),X(2),X(3),\ldots ,X(n)\}$$

(14) $$X_{i}(n)=\{X_{i}(1),X_{i}(2),X_{i}(3),\ldots ,X_{i}(n)\},i=1,2,\ldots ,n$$

This paper uses the rheological property index and chemical composition index as reference series and the molecular weight and molecular structure index as comparative series.

(2) Correlation degree calculation

The samples must be dimensionless, since the dimensions of different data samples are different, to ensure the equivalence and homogeneity of each factor:

(15) $$X_{i}^{\prime }=\{\frac{X(1)}{X(1)},\frac{X(2)}{X(1)},\frac{X(3)}{X(1)},\ldots ,\frac{X(n)}{X(1)}\}$$

(16) $$X_{i}^{\prime }(n)=\{\frac{X_{i}(1)}{X_{i}(1)},\frac{X_{i}(2)}{X_{i}(1)},\frac{X_{i}(3)}{X_{i}(1)},\ldots ,\frac{X_{i}(n)}{X_{i}(1)}\},i=1,2,\ldots ,n$$

(3) Calculate the absolute difference

(17) $$X_{0}^{0^{‘}}(n)=\{X_{0}^{0^{\prime }}(1)-X_{0}^{0^{\prime }}(1),X_{0}^{0^{\prime }}(2)-X_{0}^{0^{\prime }}(1),\ldots ,X_{0}^{0^{\prime }}(n)-X_{0}^{0^{\prime }}(1)\}$$

(18) $$X_{i}^{0^{‘}}(k)=\{X_{i}^{0^{\prime }}(1)-X_{i}^{0^{\prime }}(1),X_{i}^{0^{\prime }}(2)-X_{i}^{0^{\prime }}(1),\ldots ,X_{i}^{0^{\prime }}(n)-X_{i}^{0^{\prime }}(1)\}$$

(4) Calculate the correlation coefficient

(19) $$\left|s_{0}^{\prime }\right|=\left|\sum_{m=2}^{n-1}X_{0}^{0^{\prime }}(m)+\frac{1}{2}X_{0}^{0^{\prime }}(n)\right|$$

(20) $$\left|s_{i}^{\prime }\right|=\left|\sum_{m=2}^{n-1}X_{i}^{0^{\prime }}(m)+\frac{1}{2}X_{i}^{0^{\prime }}(n)\right|$$

(21) $$\left|s_{i}^{\prime }-s_{0}^{\prime }\right|=\left|\sum_{m=2}^{n-1}\left(X_{i}^{0^{\prime }}(m)-X_{0}^{0^{\prime }}(m))+\frac{1}{2}(X_{i}^{0^{\prime }}(n)-X_{0}^{0^{\prime }}(n)\right)\right|$$

(5) Calculate the relative correlation degree

(22) $$r_{0i}^{\prime }=\frac{1+\left|s_{0}^{\prime }\right|+\left|s_{i}^{\prime }\right|}{1+\left|s_{0}^{\prime }\right|+\left|s_{i}^{\prime }\right|+\left|s_{i-0}^{\prime }\right|}$$

According to the calculated results for the relative correlation degree, the larger the value of $r_{0i}^{\prime }$ is, the better the correlation between the comparison sequence and the reference sequence.

Some studies have suggested that a correlation between calculation indicators can be demonstrated when the correlation coefficient is ≥0.6 [33,34]. Hence, to guarantee a significant correlation between the calculated indexes, a correlation coefficient of 0.7 was employed as the threshold value to analyze the rheological properties and molecular composition of the asphalt.

### 2.7. Experiment Method

**Figure 4:**
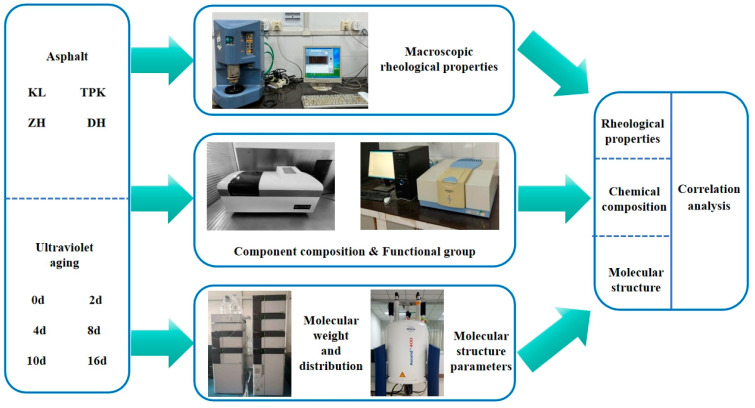
Diagram of experiment method.

To explore the impacts of ultraviolet aging on the macro and micro behaviors of asphalt and uncover the molecular mechanism underlying its rheological properties and chemical composition, four types of asphalt were chosen to investigate the UV aging times of 0 d, 2 d, 4 d, 8 d, 10 d, and 16 d. The rheological properties, four components, the characteristic peaks and functional groups, the molecular weight and molecular distribution, and the molecular structure parameters of the samples were determined through dynamic rheology, component composition, infrared spectroscopy, gel chromatography, nuclear magnetic resonance, and other tests. The influence of ultraviolet aging on the rheological properties and chemical composition of asphalt was analyzed. It was found that ultraviolet aging leads to the deterioration of the macroscopic properties of asphalt and increases its chemical stability. Based on the grey relational method, the correlation between the asphalt’s molecular composition and its macro and micro behaviors was established by calculating the degree of correlation, and it was found that behind the changes in the macro and micro behaviors of asphalt are the reactions of molecular deconstruction and reconstruction. The specific experimental scheme is presented in Figure 4.

## 3. Results and Analysis

### 3.1. Rheology Index

#### 3.1.1. Phase Angle and Rutting Factor Change

**Figure 5:**
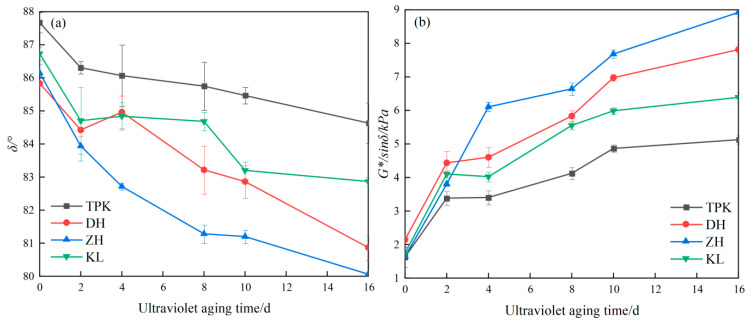
*δ* and G*/sinδ changes in asphalt under different UV aging times. (**a**) *δ* changes. (**b**) G*/sinδ changes.

The rutting factor (G*/sinδ) and phase angle (*δ*) characterize the high-temperature viscoelastic properties of asphalt, which are mainly used to describe its rutting resistance and deformation recovery ability. The larger the G*/sinδ is, the stronger the resistance to permanent deformation of the asphalt, and the smaller the *δ* is, the closer the asphalt is to being a complete elastomer. The changes in the phase angle (*δ*) and rutting factor (G*/sinδ) of the asphalt are shown in Figure 5.

The G*/sinδ of each asphalt sample after 16 d of aging increased by 214.48%, 262.75%, 442.31%, and 267.18%, respectively, compared with the original asphalt, indicating that the anti-deformation ability and elastic recovery ability of the asphalt was enhanced. At the same time, with the increase in the UV aging time, the G*/sinδ growth rate of asphalt gradually decreases, indicating that the ultraviolet aging group of asphalt occurs in the early stage, and the aging rate gradually decreases in the later stage. The *δ* of each asphalt sample after 16 d of aging decreased by 2.45%, 6.99%, 7.62%, and 4.58%, which shows that the elastic composition of the asphalt increased after aging. By comparing the changes in the G*/sinδ and *δ* of the asphalt, it can be observed that both meet the requirements of regularity and consistency. Compared with *δ*, the change value of the G*/sinδ was more significant and discriminative. Hence, G*/sinδ can be selected as an index to evaluate the UV aging rheological properties of asphalt.

#### 3.1.2. Changes in Non-Recoverable Creep Compliance and Creep Recovery Rate

**Figure 6:**
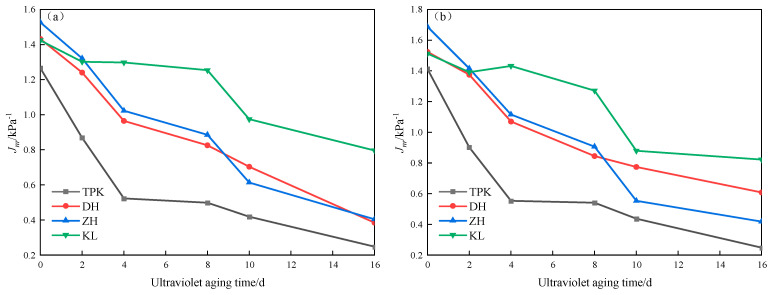
*Jnr* changes in asphalt under different UV aging times. (**a**) *J_nr_*_0.1_ changes. (**b**) *J_nr_*_3.2_ changes.

**Figure 7:**
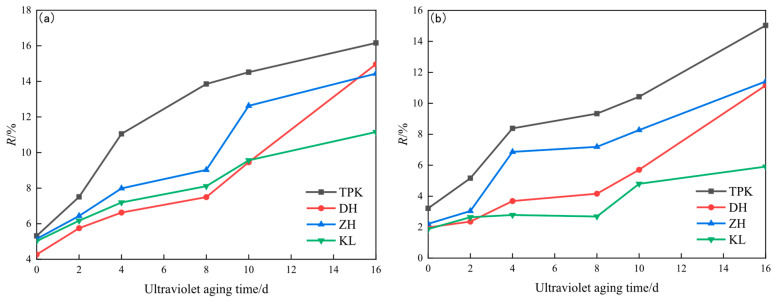
*R* changes in asphalt under different UV aging times. (**a**) *R*_0.1_ changes. (**b**) *R*_3.2_ changes.

The non-creep recovery compliance (*J_nr_*) and creep recovery rate (*R*) are mainly used to characterize the creep properties of asphalt. The smaller the *J_nr_* value is, the smaller the creep relaxation deformation of asphalt and the stronger its resistance to permanent deformation. The larger the *R*-value, the better the resilience of the asphalt. The changes in the *J_nr_* and *R* of the sample are shown in Figure 6 and Figure 7.

The *J_nr_* decreases and the *R* increases with the increase in the ultraviolet aging time of asphalt, as displayed in Figure 6 and Figure 7. This indicates that, with the deepening of the aging degree, asphalt has an enhanced resistance to high-temperature deformation and elastic recovery ability. The reason for this is that, with the gradual deepening of the aging degree, the composition of asphalt changes. After ultraviolet aging, asphalt’s content saturation and aromatic content decreases, and the content of asphaltene increases. Since the saturation and aromatic components mainly play the role of softening and lubricating asphalt, asphaltene mainly plays the role of the “skeleton”. With the deepening of the aging degree, the components that play the role of softening and lubricating decrease in number, and the components that play the role of the “skeleton” increase, finally making the asphalt develop towards the direction of being an elastomer [31]. According to the requirements of regularity, consistency, and discrimination, the *J_nr_* and *R* of the asphalt shown in Figure 6 and Figure 7 can be compared, and the change in the *R* under ultraviolet aging can be seen to be more significant than that in *J_nr_*. Therefore, *R* is selected as the index to be used in evaluating the resilience of asphalt under ultraviolet aging. Compared with *R*_0.1_, *R*_3.2_ exhibits more conspicuous alterations before and after aging; thus, *R*_3.2_ is chosen as the evaluation index.

### 3.2. Chemical Composition Index

#### 3.2.1. Component Composition Change

**Figure 8:**
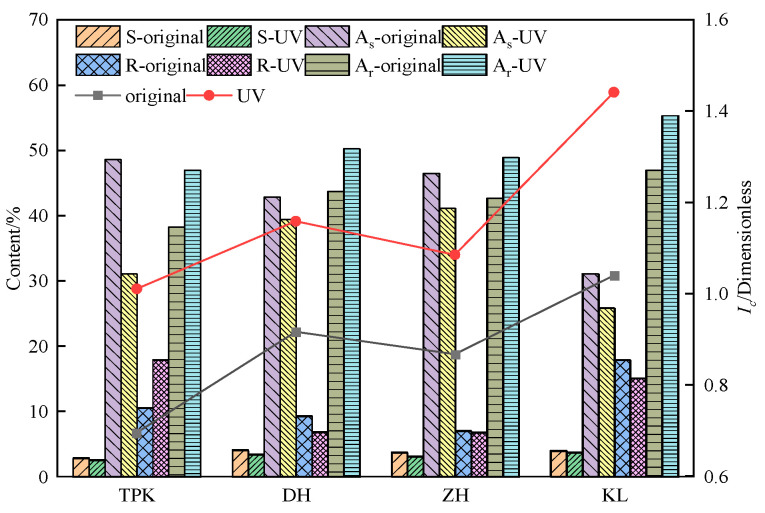
The change in four components and colloidal instability index.

The test results of four components and the *I_c_* of the asphalt before and after ultraviolet aging for 16 d are presented in Figure 8. It can be observed from Figure 8 that the light element of the asphalt decreases, and that the recombination component increases under UV aging. The reason for this is that the saturated component is mainly the mixture of straight chain and branched chain alkanes and cycloalkanes, and the aromatic component is primarily the aromatic hydrocarbon containing naphthenes and long-chain alkyls. In contrast, the straight chain and branched chain alkanes and long-chain alkyls are easy to break under the action of high ultraviolet photon energy; the free molecules, after breaking, will gradually connect to the asphaltene molecules with a relatively stable ring structure. Due to the increase in ageing time, the colloid content in TPK rose by 7.34%. Simultaneously, DH, ZH, and KL decreased by 2.45%, 0.30%, and 2.38%, respectively, due to the varying conversion rates among the aromatic components, colloid, and asphaltene during aging. The ultraviolet aging process of asphalt is the process of the conversion of light components into the gum and then into asphaltene. As the intermediate of the conversion, the amount of gum will increase if the conversion rate of light components into gum is faster than that of the gum into asphaltene; otherwise, the amount of gum will decrease.

The change in *I_c_* indicates that, after 16 d of UV aging, the *I_c_* of the TPK, DH, ZH, and KL was increased by 0.32%, 0.24%, 0.22%, and 0.40%, respectively. The outcomes demonstrate that UV aging transformed the asphalt from a sol-type to a gel-type structure. Since the gel index can comprehensively reflect the alteration of each asphalt component, Ic is chosen to assess the composition of asphalt components in accordance with comprehensiveness and discriminability.

#### 3.2.2. Characteristic Peaks and Functional Groups

(1) Characteristic peaks analysis

**Figure 9:**
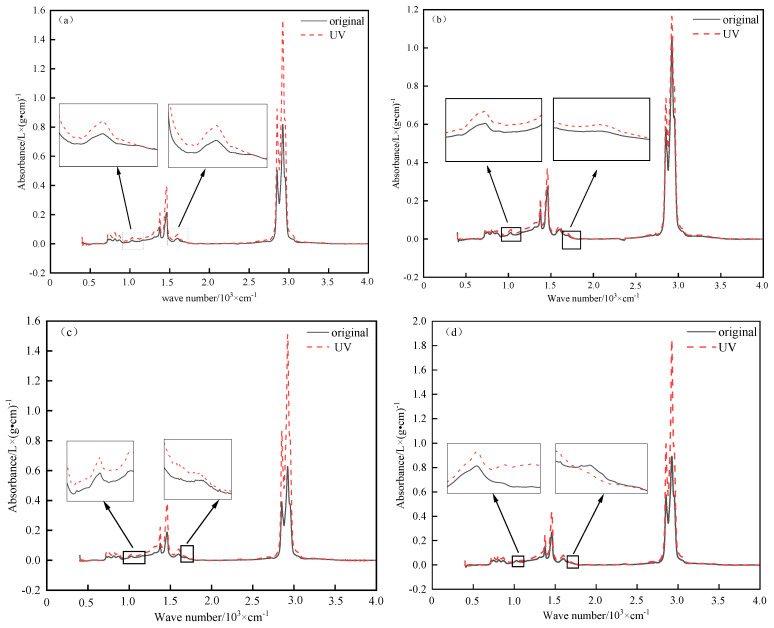
UV aging characteristic peaks of different asphalts. (**a**) TPK characteristic peaks. (**b**) KL characteristic peaks. (**c**) DH characteristic peaks. (**d**) ZH characteristic peaks.

The characteristic peaks of different asphalt samples before and after 16 d of aging are shown in Figure 9. The characteristic peaks of the infrared spectrum of various bitumen samples are different in terms of the intensity of the absorption peaks, but the location, range, and number of the characteristic peaks are the same. It can be seen from Figure 9 that the maximum absorption peaks of the asphalts before and after ultraviolet aging occur near 2924 cm^−1^ and 2852 cm^−1^, respectively, which are the stretching vibration absorption peaks of methyl —CH_3_— and methylene —CH_2_—, among which the absorption peak of methylene CH_2_— is the strongest. At the same time, the characteristic peaks around 1377 cm^−1^, 1462 cm^−1^, and 1600 cm^−1^ are also found to have obvious changes, and the changes in these peaks are mainly caused by the antisymmetric stretching vibration of —CH_2_, the stretching vibration of —CH_3_, the stretching vibration of the C=C skeleton, and the skeleton vibration of the benzene ring. Therefore, it can be determined that the —CH_3_—, —CH_2_—, —CH_2_, —CH_3_, and C=C compositions of the four bitumen samples are roughly the same. Still, some particular chemical components are slightly different in terms of their content. According to the local magnification image, new characteristic peaks appeared near 1030 cm^−1^ and 1700 cm^−1^ after ultraviolet aging, among which the characteristic peaks near 1030 cm^−1^ were caused by the stretching vibration of the alkyl or aromatic sulfoxide group (S=O). The distinctive peak near 1700 cm^−1^ belongs to the flexible vibration peak of the carbonyl group (C=O) [26,35].

(2) Functional group index analysis

**Figure 10:**
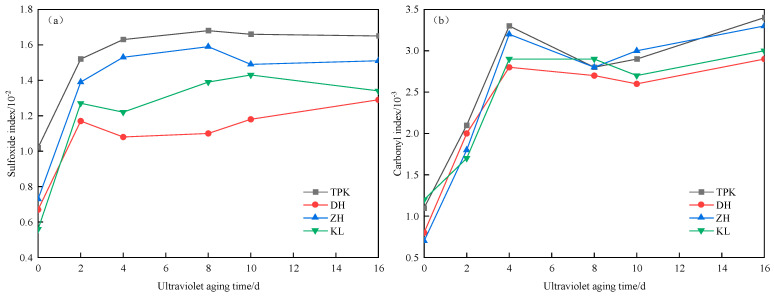
Changes in sulfonyl and carbonyl indices with UV aging time. (**a**) Sulfoxide index. (**b**) Carbonyl index.

The changes in the sulfoxide and carbonyl groups before and after 16 d of ultraviolet aging of the asphalt samples are presented in Figure 10. The sulfoxide and carbonyl indices rise with the increment of the asphalt aging time. The alterations in functional groups are more pronounced with the intensification of the aging degree. Simultaneously, the sulfone and carbonyl groups are generated due to the double bond of oxygen atoms after aging, which signifies that an oxidation reaction occurs in the ultraviolet aging of asphalt.

The sulfoxide and carbonyl groups’ index alterations exhibited a rapid increment in the initial aging stage and a stable tendency in the subsequent stage. The cause for this is that, in the early phase of ultraviolet aging, owing to the direct exposure of ultraviolet light on the asphalt surface, the chemical bonds with low energy on the asphalt surface are disrupted, leading to the reconfiguration of the internal structure of the asphalt, and the functional group index rises rapidly. As the aging time gradually increases, the ultraviolet aging of asphalt commences to progress to a deeper extent. The ultraviolet aging rate declines as the aging degree of asphalt gradually becomes saturated in the later stage. Since the amounts of both the sulfoxide and carbonyl groups increase significantly after the ultraviolet aging of asphalt, *SI* and *CI* are chosen as the evaluation indices of the internal functional groups of asphalt.

### 3.3. Molecular Composition Index

#### 3.3.1. Molecular Weight and Its Distribution Change

(1) Change in molecular weight

**Figure 11:**
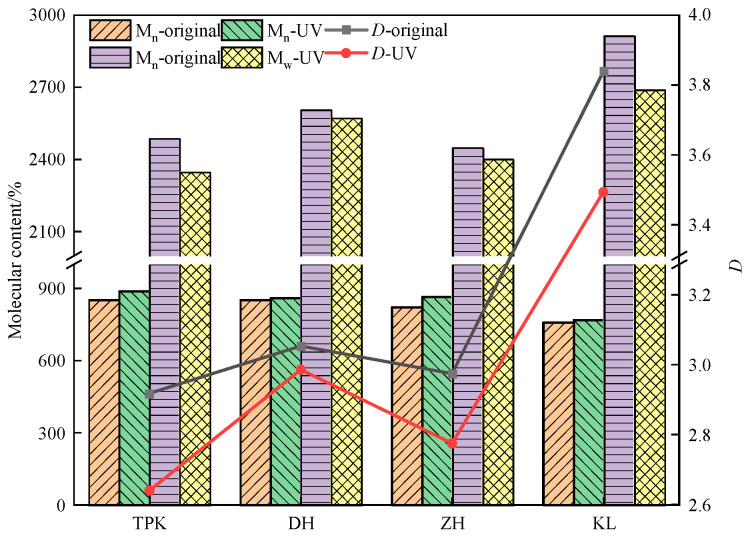
Changes in number average molecular weight (*M_n_*), weight average molecular weight (*M_w_*), and dispersion coefficient (*D*) of asphalt before and after ultraviolet aging.

The number average molecular weight (*M_n_*) is composed of homologous mixtures with the same chemical composition but different polymers, so it is mainly used to characterize the sizes of molecules. The weight average molecular weight (*M_w_*) is a mixture of homologs of different molecular weights and is the average molecular weight calculated by mass. The dispersion coefficient (*D*) indicates the degree of dispersion of the molecules inside the asphalt. The molecular weight changes before and after 16 d of ultraviolet aging of the asphalt samples are shown in Figure 11.

The maximum increase in *M_n_* for ZH is 5.10%, followed by TPK, KL, and DH. The rise in *M_n_* indicates that asphalt aging is a process in which small molecules continue to decrease and aggregate into large molecules. Ultraviolet aging will make the molecules in asphalt agglomerate. The reduction in *M_w_* for KL is up to 7.72%, followed by TPK, ZH, and DH. It can also be seen from formula (5) that the influence of large molecular weight compounds on *M_w_* is more significant than that of small molecular compounds and the increase of the combined *M_n_*. The decrease in *M_w_* is mainly due to the breaking of intermolecular chemical bonds in the original asphalt. It was found that the number and weight average molecular weight of ZH and KL changed the most. Among them, the number average molecular weight of ZH increased the most. The number average molecular weight indicates the size of molecules. At the same time, the main macromolecules in asphalt are mainly cyclic molecules of asphaltenes. The asphaltenes increase in number after ultraviolet aging [25,30], so the average molecular weight of ZH increases the most, mainly due to its high content of cyclic molecules of asphaltenes. The weight average molecular weight of KL decreases the most, and the weight average molecular weight is mainly influenced by tiny molecular compounds. The decrease in the average molecular weight indicates an increase in the number of small molecular compounds. Small and medium-sized molecules mainly come from the fracture of aromatic long-chain and multi-branched chain molecules in the ultraviolet aging process of asphalt. Hence, the average molecular weight of KL decreases the most, mainly due to its high aromatic content.

(2) Changes in the distribution of molecular relative mass.

**Figure 12:**
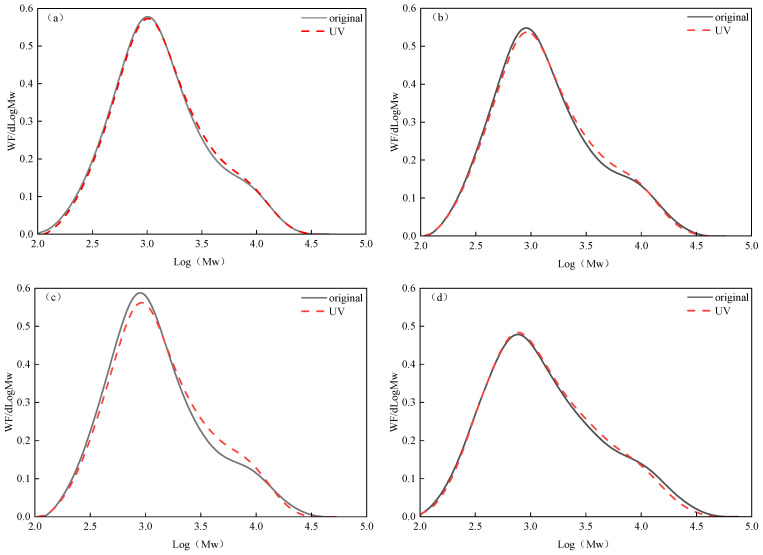
Changes in the relative mass distribution of asphalt molecules before and after ultraviolet aging. (**a**) TPK relative mass distribution. (**b**) DH relative mass distribution. (**c**) ZH relative mass distribution. (**d**) KL relative mass distribution.

The distribution of the relative quality of the asphalt samples before and after 16 d of ultraviolet aging is shown in Figure 12. It can be seen from Figure 11 that, compared with the original asphalt, the molecular relative mass distribution diagram after 16 days of ultraviolet aging shows a trend of moving from left to right, indicating that asphalt molecules generally move in the direction of macromolecules after ultraviolet aging. In the range of 10^2.0^–10^3.5^, the relative mass distribution of asphalt molecules is relatively concentrated. Because the molecular relative mass after aging is lower than that of the original, it can be seen that the molecular relative mass in this region decreases with the extension of the ultraviolet aging time. In the range of 10^3.5^–10^4.0^, acromial peaks appear in the distribution curve after ultraviolet aging, and the relative molecular mass after aging is higher than that of the original, indicating that the relative molecular mass in this region is increased, which suggests that polymerization reactions will occur between molecules with the deepening of the ultraviolet aging degree. Valley peaks appeared in the range of 10^4.0^–10^4.5^, and the molecular relative mass after aging decreased compared to the original. It can be seen that, under ultraviolet irradiation of the matrix asphalt, the chemical bond energy of the asphalt is lower than the ultraviolet photon energy, which causes the macromolecular organic compounds to break and form more minor organic compounds, and the matrix asphalt molecules are widely distributed. However, the dispersibility decreases after ultraviolet aging, which makes the small molecular compounds continue to gather into large molecular compounds. The presence of both acromion and valley peaks in Figure 12 indicates that the chemical bonds of the molecules are broken due to ultraviolet photon energy in asphalt aging, and it can be assumed that the broken free molecules will be connected to more extensive and more stable molecules, meaning that the molecular weight will both decrease and increase.

#### 3.3.2. Molecular Structure Change

(1) ^1^H-NMR spectra

**Figure 13:**
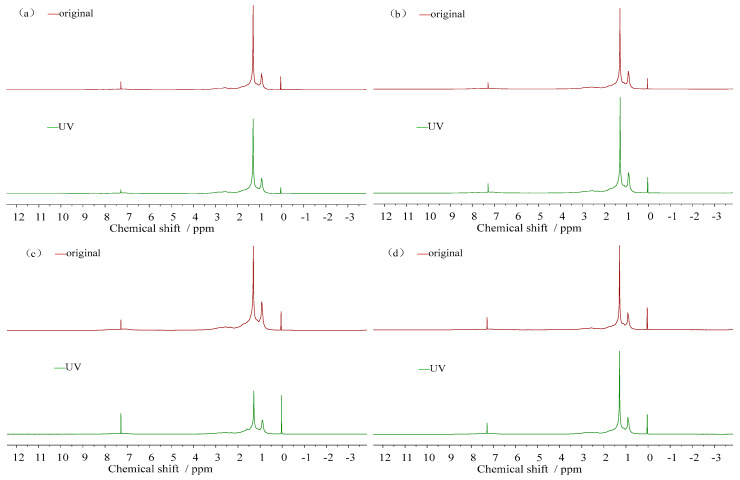
Changes in ^1^H-NMR spectra of asphalt before and after ultraviolet aging. (**a**) ^1^H-NMR spectra of TPK. (**b**) ^1^H-NMR spectra of ZH. (**c**) ^1^H-NMR spectra of KL. (**d**) ^1^H-NMR spectra of DH.

The ^1^H-NMR spectra and the chemical peak area of the 16 d ultraviolet aging nuclear magnetic resonance test for asphalt are presented in Figure 13 and Table 3. Three peaks of different intensity appeared at 0~6 ppm and 6~9 ppm, respectively, indicating that the position of the hydrogen spectrum absorption peaks of the asphalt samples was similar for each one. Still, the peak intensity was different in some fatty and aromatic regions. The peak area reached the maximum at 1~2 ppm. That of TPK and DH increased by 1.4% and 3.3% compared to before aging, while that of ZH and KL decreased by 10% and 79.2%, indicating fewer alkyl substituents in the aromatic rings of ZH and KL. TPK and DH have more types and more complicated arrangements. At the same time, it can be seen from Table 3 that the peak strength of asphalt at 1~2 ppm is greater than at 0.5~1 ppm and 6~9 ppm, indicating that the alkyl substituents in the aromatic ring of asphalt are mainly methylene, and that the content of substituted methyl groups is relatively small. This shows that the alkyl substituent length of bitumen is considerable. Approximately 7–8 ppm of asphalt is hydrogen directly connected with aromatic carbon, and the wave peak of each asphalt decreased near 7.25 ppm after aging; especially the peak of TPK decreased relatively strongly, indicating that hydrogen substitution and structural dissimilation occur in the benzene ring during the ultraviolet aging process.

(2) Change of hydrogen content

**Figure 14:**
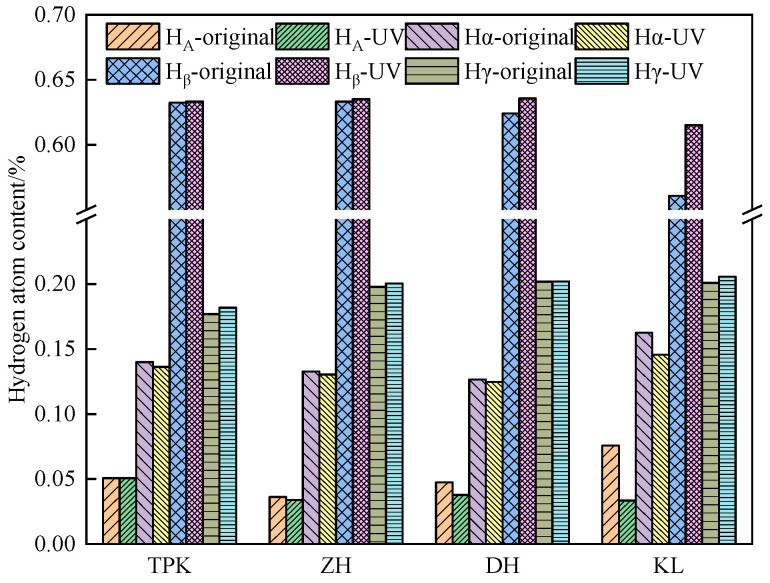
Changes in hydrogen atom content of asphalt.

The hydrogen atom contents in the asphalt samples before and after ultraviolet aging for 16 d and its change results are presented in Figure 14. The highest proportion of *H_β_* in the asphalt before and after ultraviolet aging was 55–65%, followed by *H_γ_*, *H_α_*, and H_A_, indicating that asphalt contains abundant aromatic rings and cycloalkanes. The aromatic family in the molecule mainly exists in the form of thick cyclic aromatic hydrocarbons with side chains. After ultraviolet aging, the content of *H_A_* decreased; the content in KL decreased by 25.88%, followed by DH, ZH, and TPK, indicating that the degree of condensation of the aromatic rings gradually increased during aging. After aging, the content of *H_α_* decreased, and the content of *H_β_* increased, indicating that the asphalt isomerization occurred in the process of ultraviolet aging; the change rates of the *H_α_* and *H_β_* contents were KL > TPK > ZH > DH. After ultraviolet aging, the H_γ_ content of the bitumin samples increased, among which that of TPK increased up to 2.77%, followed by KL, ZH, and DH, indicating that the alkyl substituents on the aromatic ring increased after ultraviolet aging, mainly because the long-chain fracture, cyclization, or aromatization produced more short branch chains. Since aromatic rings and aromatic hydrocarbons with side chains mainly exist in asphaltene, asphaltene, after ultraviolet aging, is primarily derived from aromatic fractions with long chains and multiple branched chains; the maximum change in KL in *H_A_, H_α_*, and *H_β_* indicates that the long chains and multiple branched chains exist and break the most, which is consistent with the results of the GPC test.

(3) ^13^C-NMR spectra

**Figure 15:**
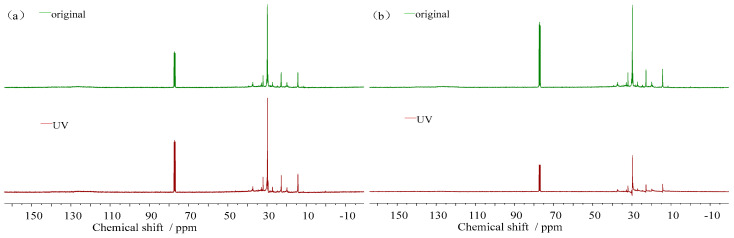

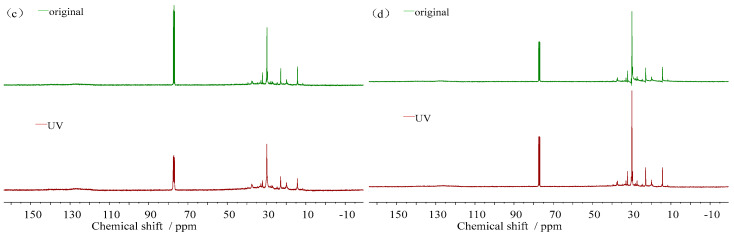
Changes in ^13^C-NMR spectra of asphalt before and after ultraviolet aging. (**a**) ^13^C-NMR spectra of TPK. (**b**) ^13^C-NMR spectra of ZH. (**c**) ^13^C-NMR spectra of KL. (**d**) ^13^C-NMR spectra of DH.

The ^13^C-NMR spectra and carbon atom content of the 16 d ultraviolet aging nuclear magnetic resonance test of the asphalt samples are presented in Figure 15 and Table 4. The *C_A_* and *C_N+P_* of the asphalt increased, thereby causing the *f_A_* to increase, and the change rates of both indexes were DH > TPK > ZH > KL. The reason for this is that aromatic carbon exists in the form of an aromatic ring, which is a highly stable structure, and only a higher energy can play the role of ring opening. In contrast, fatty carbon exists in the form of a chain. With the increase in the ultraviolet aging degree and carbon chain length, the C=C bond energy in the carbon chain is gradually weakened, enabling the fatty carbon to be connected to the aromatic structure in the form of substituents. Thus, the aromatic carbon content in the asphalt increases, while the content of fatty carbon decreases. This is also in line with the analysis of the nuclear magnetic resonance hydrogen spectrometry of the samples. Simultaneously, since the aromatic structure typically exists on the polymer main chain, the increase in the aromatic structure will make the internal rotation of the molecular main chain difficult, resulting in an increase in the rigidity of the aromatic structure and a decrease in the rheological property of the asphalt.

(4) Molecular structure parameters

**Figure 16:**
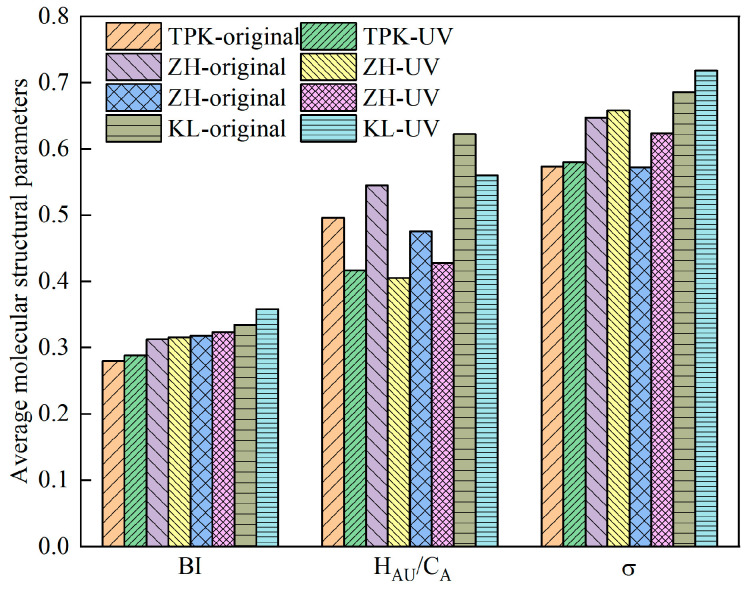
The condensation index of the aromatic ring system (*H_AU_*/*C_A_*), the degree of branching of the alkane chain (*BI*), and the peripheral hydrogen substitution rate (*σ*).

Figure 16 presents the average molecular structure parameters of the asphalt samples before and after ultraviolet aging. The *BI* increased after ultraviolet aging, and KL had the largest increase at 7.12%, followed by ZH, TPK, and DH. Because *BI* reflects the differentiation of alkanes, the greater the value of the alkyl partial branch chain or the lower the number of naphthenic chains. The *H_AU_*/*C_A_* decreased after UV aging, and the maximum value, that of ZH, was 25.60%, followed by TPK, DH, and KL, indicating that UV radiation caused the chemical bond between high-polymer molecules to break. At the same time, the *σ* also increased after ultraviolet aging; that of TPK increased by up to 3%, followed by KL, DH, and ZH. The increase in *σ* indicates that the number of alkyl branch chains in asphalt increases, which further suggests that ultraviolet aging will lead to the breaking of chemical bonds in asphalt.

### 3.4. Molecular Structure Analysis of Rheological Properties and Chemical Composition

The rheological properties and chemical structure of asphalt are influenced by the alteration of the molecular composition within the asphalt, which is the macroscopic manifestation of changes in molecular weight and molecular structure. Consequently, the relationship between UV aging and rheological properties was investigated based on the molecular weight and structure parameters of asphalt. X_01_~X_05_ was defined by taking the G*/sinδ, *R*_3.2_, *I_C_*, *CI*, and *SI* as reference series. Taking the M_n_, *f_A_*, *BI*, *H_AU_*/*C_A_*, and *σ* as comparative series, X_1_~X_5_ was defined. The correlation degree of the series was calculated, and the results are shown in Table 5.

The G*/sinδ has the most significant correlation with the number average molecular weight, indicating that the influence of the molecular weight is the greatest. The *R* has the most significant correlation with the aromatic carbon rate and the branching degree of the alkyl chain rate, indicating that the *R* is affected by the branching chain and aromatic structure of the alkyls and naphthenes.

The strong correlation between the *I_C_* and the carbon content of aromatics suggests that the cyclic aromatic structure significantly influences the *I_C_*. The functional group index, represented by the *SI* and *CI* groups, is highly correlated with all molecular structure indexes, indicating that any alteration in the molecular structure after aging will impact the number of functional groups.

## 4. Discussions

From the aforementioned studies, it is evident that the ultraviolet aging of asphalt is primarily influenced by alterations in the chemical and molecular composition of asphalt, while the rheological property is merely its macroscopic manifestation. It has been discovered that the essence of the ultraviolet aging of asphalt is oxidation. Oxygen in the air reacts with hydrogen, carbon, and other elements in asphalt under the excitation of ultraviolet light, thereby enhancing the molecular backbone stiffness of asphalt. Besides oxidation, the high photon energy of ultraviolet light also assumes a crucial role in aging. The high photon energy of ultraviolet light breaks the long-chain and branched-chain molecules in the asphalt, and the generated free molecules will be linked to the stable aromatic ring, leading to an increase in the number of macromolecules in the asphalt and an improvement in its stability. It can be concluded that the oxygen content and the quantity and weight of the aromatic ring structure are the most significant controlling factors in the ultraviolet aging sensitivity of asphalt, and the generation and development of the ultraviolet aging of asphalt are controlled and explained, respectively, by the oxidation and light energy. From the above, it can be concluded that the future research direction of asphalt UV aging should mainly focus on the chemical composition and molecular structure of asphalt. Meanwhile, since the analysis of the microscopic test results in this paper is mainly qualitative and semi-quantitative, subsequent studies can further determine the chemical composition and molecular structure of asphalt under UV aging through accurate quantitative research.

## 5. Conclusions

In this paper, the properties of asphalt after ultraviolet aging were analyzed in terms of its rheological properties, chemical composition, and molecular structure. On this basis, the rheological properties and chemical composition of asphalt after aging were characterized by correlation analysis. The main findings are summarized as follows:

(1) After UV aging, the anti-deformation, anti-high-temperature deformation, and elastic recovery capacity of asphalt are enhanced, while the flow performance is reduced. It can be observed that the internal elastic components of asphalt increase and transform into gel after aging;
(2) Under the influence of ultraviolet light, the carbon and sulfur molecules within the asphalt will combine with oxygen elements to form stable chemical functional groups and increase the molecular weight of the asphalt. This indicates that the essence of the ultraviolet aging of asphalt is oxidation;
(3) Ultraviolet aging leads to a reduction in *H_A_*, for which the decrease in KL was 55.88%, suggesting that hydrogen atoms in the benzene ring of asphalt are substituted and isomerized. This demonstrates that the dehydrogenation of asphalt takes place during the aging process;
(4) After 16 days of aging, the aromatic carbon of TPK, DH, ZH, and KL increased by 34.23%, 49.74%, 28.91%, and 12.63%, respectively. This shows that the aromatic structure of asphalt is changed and the molecular stability is enhanced after ultraviolet aging;
(5) The correlation analysis conducted herein shows that the correlation coefficients between sulfoxide group, carbonyl group, and molecular structure indexes are between 0.7–0.98, indicating that oxidation is the essence of UV aging and that the effect of ultraviolet light is mainly excitation.

The aging effect of ultraviolet radiation on asphalt essentially changes the molecular structure of asphalt through high photon energy so that the internal molecules of the asphalt are deconstructed and reconstructed. The macro and micro indexes of asphalt are only the embodiment of the internal molecular changes. Therefore, it is necessary to further study the molecular structure of asphalt, understand the changes in and mechanisms of asphalt molecules, and, on this basis, establish a numerical model of the changes in asphalt molecules under ultraviolet aging by means of mathematical simulation.

## Figures and Tables

**Table 1:** Physical properties of asphalt.

Asphalt Types	Penetration (25 °C, 0.1 mm)	Softening Point/°C	Elongation (15 °C)/cm
TRK	75.0	47.5	83.2
DH	70.0	48.1	75.3
ZH	65.0	49.4	62.4
KL	67.0	48.7	70.1
Technical requirement	60.0~80.0	≥43.0	≥40.0
Test method	T0604	T0606	T0605

**Table 2:** Attribution of hydrogen atoms and carbon atoms.

Atom	Area	Chemical Shift (δ)/ppm	Attribution of Atom
Hydrogen atom	*H_A_*	6.0~9.0	Hydrogen is directly linked to the aromatic carbon
	*H_α_*	2.0~4.0	Hydrogen attached to the α carbon of the aromatic ring
	*H_β_*	1.0~2.0	Hydrogen on the β carbon of the aromatic ring and hydrogen on the CH_2_ and CH groups beyond β
	*H_γ_*	0.5~1.0	Hydrogen at the γ position of the aromatic ring and the CH_3_ group from γ away
Carbon atom	Aromatic carbon	150~170	Aromatic carbon associated with an -OH or -OR
		130~150	Aromatic carbon or Aromatic ring carbon associated with -R
		100~130	Aromatic carbon associated with -H
	Fatty carbon	14.1	CH_3_—(CH_2_)_n_— n ≥ 3
		19.7	—CH_2_—CH (CH_3_)—CH_2_—
		22.7	CH_3_—CH_2_—(CH_2_)_n_— n ≥ 2
		29.7	CH_3_—CH_2_—CH_2_—(CH_2_)_n_—CH_2_—CH_2_—
		32.0	CH_3_—CH_2_—CH_2_—(CH_2_)_n_— n ≥ 2

**Table 3:** Chemical peak area of asphalt hydrogen spectrogram.

Asphalt Type	Aging Condition	0.5~1 ppm	1~2 ppm	6~9 ppm	7~8 ppm
TPK	original	0.2039	0.2796	0.4960	0.5798
	UV	0.2310	0.2880	0.4164	0.5732
ZH	original	0.1634	0.3125	0.5445	0.6470
	UV	0.2020	0.3155	0.4051	0.6579
DH	original	0.1963	0.3233	0.4756	0.5718
	UV	0.1955	0.3176	0.4275	0.6232
KL	original	0.0811	0.3344	0.6222	0.6855
	UV	0.1317	0.3582	0.5600	0.5180

**Table 4:** Carbon atom distribution parameters.

Asphalt Type	Aging Condition	C_A_	C_N+P_	*f_A_*
TPK	original	12.2251	45.3986	0.2122
	UV	16.4098	44.1438	0.2710
DH	original	8.0504	51.8002	0.1345
	UV	12.0555	47.2284	0.2034
ZH	original	9.6214	43.6227	0.1807
	UV	12.4025	40.2156	0.2357
KL	original	12.8250	46.8221	0.2150
	UV	14.4445	47.3007	0.2339

**Table 5:** Table of correlation index values.

Index	X_01_	X_02_	X_03_	X_04_	X_05_
X_1_	0.78	0.62	0.55	0.70	0.71
X_2_	0.58	0.70	0.70	0.77	0.76
X_3_	0.57	0.74	0.66	0.82	0.76
X_4_	0.57	0.61	0.59	0.90	0.82
X_5_	0.56	0.63	0.60	0.93	0.92

## Data Availability

Data will be made available on request.
